# Complete Genome Sequences of Five Human Coronavirus NL63 Strains Causing Respiratory Illness in Hospitalized Children in China

**DOI:** 10.1128/MRA.01597-19

**Published:** 2020-02-20

**Authors:** Lu Zhang, Mian Gan, Zhaoyong Zhang, Xin Li, Wenkuan Liu, Airu Zhu, Jing Sun, Fang Li, Yanqun Wang, Fuchun Zhang, Jingxian Zhao, Rong Zhou, Jincun Zhao

**Affiliations:** a Institute of Infectious Disease, Guangzhou Eighth People’s Hospital of Guangzhou Medical University, Guangzhou, China; b State Key Laboratory of Respiratory Disease, National Clinical Research Center for Respiratory Disease, Guangzhou Institute of Respiratory Health, The First Affiliated Hospital of Guangzhou Medical University, Guangzhou, China; DOE Joint Genome Institute

## Abstract

We report the complete genome sequences of five human coronavirus NL63 (HCoV-NL63) strains obtained using next-generation sequencing. The five HCoV-NL63 strains were obtained from hospitalized children with severe acute respiratory infection detected in Guangdong, China. This study provides several complete genomes of HCoV-NL63 and improves our understanding of HCoV-NL63 evolution in China.

## ANNOUNCEMENT

Human coronavirus NL63 (HCoV-NL63) is a member of the family Coronaviridae, genus Alphacoronavirus, and was first discovered in 2004 (1). HCoV-NL63 is mainly associated with the common cold in children, the elderly, and immunocompromised patients (2, 3). The genome of HCoV-NL63 is about 27 kb with a conserved gene order of 5′-orf1ab-spike (S)-orf3-envelope (E)-membrane (M)-nucleocapsid (N)-poly (A). The species tropism of HCoV-NL63 is determined by spike glycoprotein. HCoV-NL63 and severe acute respiratory syndrome coronavirus (SARS-CoV) share the same cell receptor, angiotensin converting enzyme 2 (ACE-2) (4, 5), for entry into host cells, and HCoV-NL63 is recognized as a common cause of upper respiratory tract infection and has been prevalent worldwide.

**FIG 1:**
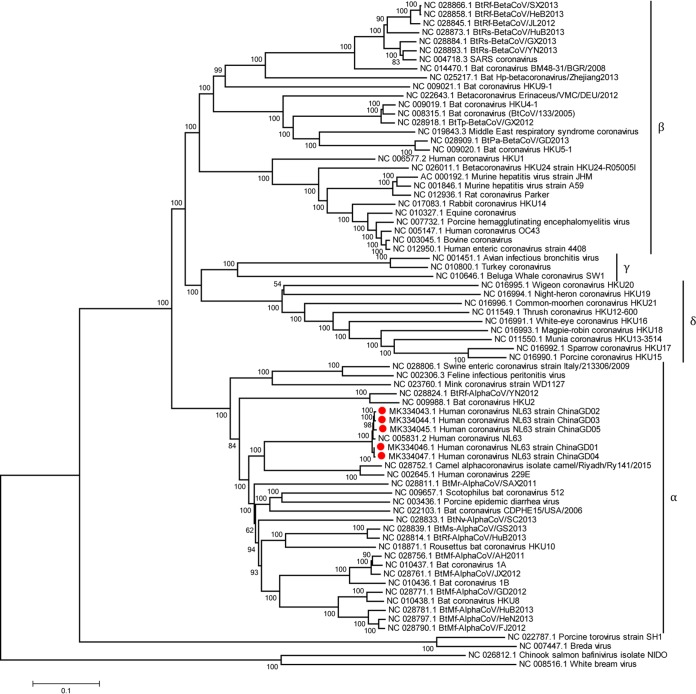
Phylogenetic analysis of HCoV-NL63 based on complete genomes. The complete genomes of 69 coronavirus references were obtained from the GenBank database, and multiple alignments were performed using MAFFT version 7.158 with default parameters. The phylogenetic tree was constructed using the neighbor-joining method with 1,000 bootstraps in MEGA version 5.10 with default parameters. The numbers at the nodes represent bootstrap support. Bootstrap values greater than 70% were considered statistically significant for grouping. The HCoV-NL63 strains presented in this study are marked with red circles.

Here, nasopharyngeal swab samples were collected from hospitalized children with severe acute respiratory infection in Guangzhou, China, in 2018. This study was performed in strict accordance with human subject protection guidance provided by the Research Ethics Committee of Guangzhou Medical University. The respiratory samples were filtered with 0.22-μm filters, RNA extraction was performed using a Qiagen viral RNA extraction kit, and extracted RNA was used for sequence-independent single-primer amplification (SISPA) (6, 7) as follows: a reverse transcription reaction was performed with SuperScript III reverse transcriptase using a primer containing a fixed sequence, followed by a random hexamer at the 3′ end (FR26RV, GCCGGAGCTCTGCAGATATCNNNNNN). Then, Klenow fragment polymerase (New England Biolabs) was used for DNA synthesis. Finally, PCR amplification was conducted using primers consisting of the fixed portions of the random primers (FR26, GCCGGAGCTCTGCAGATATC). Purified DNA was used for next-generation sequencing (NGS). Libraries were prepared with the Nextera XT kit (Illumina), and paired-end reads (2 × 125 bp) determined using a HiSeq 2500 instrument were used for cleaning and assembling using CLC Genomics Workbench version 11.0. Illumina sequencing yielded about 10 million reads per sample. Reads were assembled into contigs with a *de novo* assembly model, and the contig sequences were then extracted for subsequent analysis. Partial genome sequences of five HCoV-NL63 strains were obtained by NGS methods. Meanwhile, sets of specific primer pairs were designed and used to amplify the gap region of HCoV-NL63, which was used for genome assemblies using the SeqMan subprogram of the DNAStar software version 7.1.0 with default parameters (Table 1). Finally, five complete genome sequences of HCoV-NL63 were obtained using next-generation sequencing and Sanger sequencing methods together and were designated strains ChinaGD01 (27,531 bp), ChinaGD02 (27,516 bp), ChinaGD03 (27,516 bp), ChinaGD04 (27,532 bp), and ChinaGD05 (27,544 bp). The five HCoV-NL63 strains presented here were aligned using MAFFT version 7.158 (8) and showed 98.5 to ∼99.1% nucleotide homology with the prototype HCoV-NL63 virus (GenBank accession number NC_005831.2) as estimated using MEGA version 5.10 software (9) (Fig. 1).

**TABLE 1:** Primers used for the genome sequencing of HCoV-NL63

Primer	Sequence (5′–3′)	Target (nucleotide position) or amplification method	Size (bp)
1F	CCTGGCCTCTTGCTTTTTCACATGT	20504	1,686
1R	ACTTCGACGGTTGAGAAACAAATAG	22189	1,686
2F	CGCGTTAAGAGTGGTTCACCAGGTG	22059	1,623
2R	CAAAGCTGCAAGCCGTCCAGTAATT	23681	1,623
3F	TTCAATTCAAGCCGATCAACAAGTT	23624	1,600
3R	GTCATCAATTAATCGAAGGAACATC	25223	1,600
4F	CGAAGAGCCTGTTGTTGGTATAGTC	25153	1,690
4R	AACACGCTTCCAACGAGGTTTCTTC	26849	1,690
5F	CCAGGGCTGATAAGCCTTCTCAGTT	26800	754
5R	GTGTATCCATATCAAAAACAATATC	27553	754
6F	TGAGGATGTTTGTGTTTGTTTTGAC	18864	1,710
6R	GTCAGGAACACCTAATTGTAACATA	20573	1,710
7F	TGCGTGGTTGGTTGGGTATGGATGT	17234	1,682
7R	ACGCTCATACGAACCCTGAATACTA	18915	1,682
8F	ATTCAGCAACTGGTTCCTTAGATGT	15479	1,808
8R	GTTATCGCCACAAACATGAGCACTT	17286	1,808
9F	CTCCCTACTATGACACAGCTGAATC	14005	1,596
9R	AGCCGCAAAGAGTCTAAGTGTATCT	15600	1,596
10F	GACCGTACAACTATTCAAAGTGTTG	12398	1,659
10R	GTTCTTTACCACTAATAGCATACTT	14056	1,659
11F	GGGCTATGGCTAATGGTTATACAAG	9801	1,435
11R	TTTGCGATATTCATGGCACGCTTCA	11235	1,435
12F	ACCCTTCAGAGTGTTGCTTCATCAT	11090	1,378
12R	AGTCGAGCTGCACTAGAACCCCTTG	12467	1,378
13F	CAACCACTGTAACTAGCTTTCATGG	7758	2,103
13R	CTGCCAAAATAGAATAGCACTCAAC	9860	2,103
14F	GTCAAAAGGGTGATGCTGAAGAGGC	5424	2,394
14R	TCAACTGACCATTCTCAATGTACTT	7817	2,394
15F	TAGAGATGAATTGGGTGTTCGTGTT	3424	2,043
15R	GGTCCAACATCACCTGTAACAAATT	5466	2,043
16F	GCAGATGTTCCAGATGCTTTTCAAT	1637	1,864
16R	GCAACTGTACAAGTGTGGTACTAAT	3500	1,864
17F	CAGCAATTATGTTCTTCAGGACTTT	565	1,118
17R	GTGTAAATGTGCGATAAACTGATTG	1682	1,118
18F	CTTAAAGAATTTTTCTATCTATAGA	1	1,056
18R	CATGCACCAACACTCCAACTCTCAG	1056	1,056
GSP 1	CGAAGAGCCTGTTGTTGGTATAGTC	3′ RACE^*a*^	Unknown
AP	GGCCACGCGTCGACTAGTACTTTTTTTTTTTTTTTTT	3′ RACE	Unknown
GSP 2	CCAGGGCTGATAAGCCTTCTCAGTT	Nested PCR	Unknown
AUAP	GGCCACGCGTCGACTAGTAC	Nested PCR	Unknown
AAP	GGCCACGCGTCGACTAGTACGGGGGGGGGG	5′ RACE	Unknown
GSP 1	GTGTAAATGTGCGATAAACTGATTG	5′ RACE	Unknown
AUAP	GGCCACGCGTCGACTAGTAC	Nested PCR	Unknown
GSP 2	CATGCACCAACACTCCAACTCTCAG	Nested PCR	Unknown
GSP3	CCATGGCCAAAAACAACATCAAAGT	Nested PCR	Unknown

a RACE, rapid amplification of cDNA ends.

Only two complete genome sequences of HCoV-NL63 associated with acute respiratory illness have been obtained and reported in China. The complete genome sequence data from our study will provide insight into the evolution and genetic diversity of HCoV-NL63 in China.

### Data availability.

The complete genome sequences of the five newly identified HCoV-NL63 strains have been deposited in GenBank under the accession numbers MK334043, MK334044, MK334045, MK334046, and MK334047. The sequencing reads are available in the SRA database under BioProject accession number PRJNA601331.
